# Gun violence in Colombia 

**DOI:** 10.2471/BLT.19.021119

**Published:** 2019-11-01

## Abstract

A city in Colombia tries to address the root causes of gun violence. Sophie Cousins reports.

Fabián Parra was 11 years old when he joined a street gang. That was in 2011. The gang dealt drugs and sometimes had to defend its territory with guns. The territory was a small part of Comuna 18, a neighbourhood in Cali, Colombia, a city of 2.3 million people. 

“When I was 16, a kid from another gang tried to shoot me,” Fabián says. “I fired back and hit him in the foot. Afterwards his friends came looking for me. They were going to kill me, so I had to leave the neighbourhood.”

The year Fabián left Comuna 18, it was already becoming a safer place. According to police records there were only two gang-related homicides there in 2016, down from 11 in 2015.

The decline in the Comuna 18 homicides was part of a city-wide trend established in 2013. Before that year, Cali had about 2000 homicides per annum. In 2018 there were 1209. That still represents around 50 murders per 100 000 people, which is twice the rate reported for Medellín, a comparable Colombian city, but it is a significant fall nonetheless.

Dr María Isabel Gutiérrez, a professor of epidemiology at the School of Public Health at the Universidad del Valle in Cali, Colombia, credits the *Tratamiento Integral a Pandillas – Jóvenes sin Fronteras* (TIP, Comprehensive Treatment Program for Gangs, Youth Without Frontiers), as one reason for the decline.

A collaboration between the metropolitan police and Universidad del Valle’s *Instituto de Investigación y Desarrollo en Prevención de la Violencia y Promoción de la Convivencia Social *(Cisalva, Institute for Research and Development in Violence Prevention and Promotion of Social Coexistence), the TIP programme has been providing comprehensive psychosocial support for around 1400 youths from eight of Cali’s most violent neighbourhoods, including Comuna 18, since 2015.

“Youths come into the programme voluntarily, referred by police trained in community relations, or by one of the 56 community leaders employed by the programme,” Gutiérrez, who is one of the programme’s founders, explains.

“We work with these young men and women using an integrated psychosocial approach including individual counselling, health care and access to addiction treatment where this is required. But we also work at the family and community level, help youths complete their education and find work, and give them opportunities to play sports, and participate in music and theatre.”

When youths are judged to be at risk of quitting the programme or get into trouble, the Cisalva and police team spends more time with them in their neighbourhood. “It requires a lot of commitment, but the programme really works,” says Gutiérrez.

Since 2015 there has been an 80% decline in homicides in the eight Comunas targeted, murders dropping from 396 in 2015 to 81 in 2018. “I’ve been involved in this type of work for a long time and I have never seen such a reduction in homicides,” Gutiérrez adds.

“It is imperative that we directly engage the young people involved in violence.”

María Isabel Gutiérrez

Not everyone is convinced that a programme focused on treating individuals is the best approach to tackling gun violence. “It may be possible to change an individual’s behaviour or lifestyle, but the evidence suggests that population-wide initiatives are more effective,” says Dr Alexander Butchart, a violence prevention expert at the World Health Organization in Geneva, Switzerland.

At the top of Butchart’s list of effective prevention initiatives is the implementation and enforcement of laws that restrict access to firearms. “Several studies show that ease of access to guns is an important driver of gun violence,” he says, citing recent studies of firearm and non-firearm homicide in South African cities, which demonstrate a close association between the intensity of efforts to enforce that country’s firearms control act, and trends in firearm-related homicide rates.

While there may be compelling arguments for focusing on gun control in Colombia, it would be difficult to overstate the size of the challenges faced, including the number of unregistered firearms in circulation (around 4.2 million according to a recent estimate published by the Graduate Institute of International and Development Studies, Geneva) and the lack of effective law enforcement in much of the country.

As daunting as the challenges may be, gun control has already been tried in Colombia. In Cali, in 1993, mayor Rodrigo Guerrero, banned the carrying of guns on weekends and holidays. The ban was enforced with random pat-downs and searches at checkpoints.

“The ban was launched in response to the data generated by a fatal injury surveillance system, which was instituted by the mayor and collected data from a range of institutions including the police, hospitals and the district attorney’s office,” explains Dr Andrés Villaveces, an epidemiologist at the National Center for Injury Prevention and Control, Centers for Disease Control and Prevention in Atlanta in the United States of America.

Among the findings generated by the system was the that men between 15 and 25 were most likely to be involved in fatal injuries, 80% of deaths were caused by firearms, roughly a quarter of the victims were intoxicated, and that most murders occurred over the weekend with payday weekends being particularly lethal.

“In Cali, paydays fall at the end of every month or every fortnight; the surveillance revealed a significant spike in violent deaths on the weekends following them,” Villaveces explains.

The firearm ban was introduced along with a ban on the sale of alcohol after 01:00 during the week and 02:00 Fridays and Saturdays. According to Villaveces, the firearm ban was associated with a 14% reduction in homicides in Cali between 1993 and 1994.

As effective as Guerrero’s initiative may have been, like the TIP programme, it was only implemented at the municipal level. Jerónimo Castillo, a security and criminal policy expert at the *Fundación Ideas para la Paz* (FIP, Ideas for Peace Foundation), a research foundation based in the capital, Bogotá, believes that the restriction of Colombia’s anti-gun violence initiatives to municipal programmes is one of the reasons the country continues to experience high rates of violent deaths.

According to the FIP, after a steady decline in homicides between 2000 and 2017, with violent deaths reaching their lowest number (11 381) in 42 years in 2017, there was a 4% increase in 2018, with 12 311 deaths reported nationwide.

Castillo points to numerous drivers of this negative trend, including porous borders (which facilitate the flow of illegal arms), and a post-conflict context in which diverse criminal groups are emerging, including former members of the *Fuerzas Armadas Revolucionarias de Colombia* (Revolutionary Armed Forces of Colombia) and the *Ejército de Liberación Nacional* (National Liberation Army).

“Crime prevention policies have been developed by local mayors, but we need a national strategy based on reliable data to really tackle the problem of gun control,” Castillo says.

Castillo believes that the lack of reliable national data not only makes policy formation difficult, it gives rise to unhelpful policy biases, which can in turn lead to misallocation of resources. “For example, there is too much focus placed on criminal gangs, when in cities such as Bogotá they account for just over half of homicides,” he says. “And while that half is obviously very important, it is not the whole story.”

“We need a national strategy based on reliable data.”

Jerónimo Castillo

For Castillo, the whole story needs to include not just the gangs, but the socioeconomic circumstances that give rise to them. “The fact that the highest urban homicide rates tend to be reported in the poorest neighbourhoods is not a coincidence,” he says.

Addressing urban poverty was central to mayor Guerrero’s other important anti-violence initiative, *Territorios de Inclusión y Oportunidades* (TIO, Territories of Inclusion and Opportunities), which was launched in Cali in 2012. Targeting the city’s 11 poorest districts, TIO focused on improving schools, housing, health care and transport. The fact that the current mayor of Cali is continuing the programme suggests that its value has been noted.

Gutiérrez believes that the TIO and TIP programmes are complementary. “It is vital to invest in the urban fabric to counter disenfranchisement and marginalisation,” she says. “Violence is driven by multiple factors and we have to try to address them all, but it is imperative that we directly engage the young people involved in violence if we really want to make a change.”

The TIP programme directly engaged Fabián Parra when he returned to his neighbourhood. “I really got involved in different activities as a joke at first,” he says. “Then I started writing and singing rap songs and acting too. It completely changed my life.”

With the support and encouragement of the TIP team, Fabián also went back to school. He is currently studying for his high school diploma and is hoping to study communications at the Universidad del Valle.

Gutiérrez points out that it is not just Fabián’s life that has changed, the neighbourhood around him has changed also. “Fabián has become a role model for a lot of the kids who face the same challenges he did,” she explains. “Changing one person changes many others.”

**Figure:**
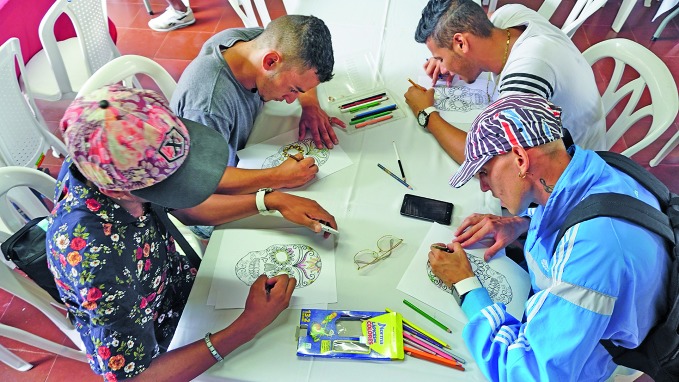
Young men decorate masks as part of a *Jóvenes sin Fronteras* characterisation activity

Cisalva Institute Communications

**Figure:**
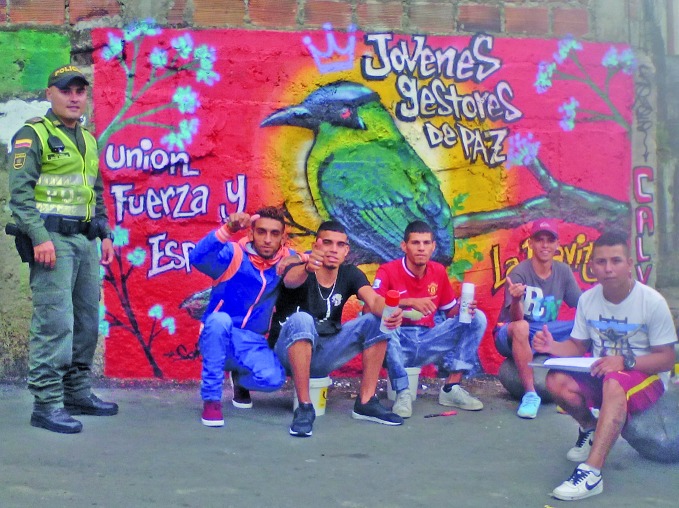
Young men accompanied by a police officer in front of a *Jóvenes sin Fronteras* mural

Cisalva Institute Communications

